# The detoxification paradox: Rethinking microbial dehalogenation in environmental remediation

**DOI:** 10.1016/j.eehl.2025.100190

**Published:** 2025-10-03

**Authors:** Qian Wang, Shuai Wang, Yueli Hu, Jiaxin Wu, Shanyuan Niu

**Affiliations:** a School of Environment, Nanjing University, Nanjing 210023, China; b College of Engineering and Applied Sciences, Nanjing University, Nanjing 210023, China

**Microbial dehalogenation, central to bioremediation, frequently falls short of achieving complete detoxification, often yielding mobile and toxic intermediate compounds—a critical oversight we term as the “detoxification paradox”. This commentary analyzes the mechanistic basis of this paradox and questions the conventional reliance on pollutant removal alone as a performance metric. We advocate for a paradigm shift toward active biological design, outlining a novel, multi-level engineering framework spanning molecular, cellular, and system scales to steer microbial processes toward genuine detoxification and ensure safer environmental remediation.**

## 1 The detoxification paradox: When microbial degradation worsens pollution

Halogenated organic pollutants (HOPs), including poly- and perfluoroalkyl substances (PFAS), polychlorinated biphenyls (PCBs), polybrominated diphenyl ethers (PBDEs), and chlorinated solvents, pose a persistent and pervasive threat to global ecosystems and human health [1,2]. Bioremediation is often promoted as a sustainable alternative to energy-intensive physicochemical methods, yet this opinion arises from an oversimplified assumption: that microbial degradation inherently detoxifies. For years, the conventional paradigm suggested that microbial dehalogenation results either in complete mineralization or the formation of simpler, less toxic compounds. However, emerging evidence reveals that microbial transformations are often incomplete, giving rise to transient intermediates that are likely to be more mobile, bioavailable, and equally if not more toxic than the parent compound. This is clearly observed with chlorinated ethenes and certain PFAS, where partial degradation produces carcinogens like vinyl chloride or persistent short-chain fluorinated acids [3,4]. This contradiction—where a process intended to detoxify instead generates hazardous products—constitutes what we term the “detoxification paradox”.

**Fig. 1:**
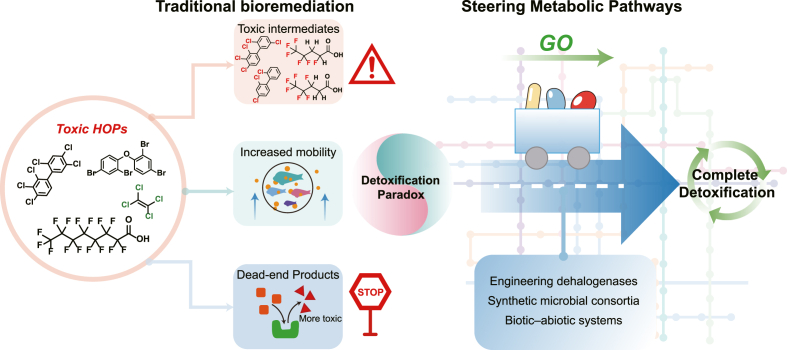
From blind degradation to precision detoxification: Resolving the microbial dehalogenation paradox.

This paradox necessitates a fundamental shift from passive observation to active biological design. Simply measuring pollutant removal is an insufficient metric for success; achieving genuine detoxification requires a paradigm shift grounded in engineering principles. To address this challenge, a multilevel framework is required, which integrates advanced strategies across molecular, cellular, and system levels to precisely regulate metabolic pathways, prevent the accumulation of toxic intermediates, and ensure effective detoxification (Fig. 1). This commentary elaborates on the mechanistic basis of the paradox and substantiates the proposed framework as a necessary advancement toward reliable and safe bioremediation.

## 2 Unraveling the paradox: Pathways to hazardous outcomes

Microbial dehalogenation occurs mainly through two distinct mechanistic pathways: anaerobic reductive dehalogenation and aerobic oxidative dehalogenation. Although each pathway employs a unique catalytic mechanism, both frequently fail to achieve full mineralization, instead accumulating products more hazardous than the original compounds.

Anaerobic reductive dehalogenation facilitates HOPs as terminal electron acceptors and is predominant for degrading highly halogenated compounds, notably PCBs and chlorinated ethenes such as perchloroethylene (PCE) and trichloroethylene (TCE). This reductive pathway is highly sensitive to environmental conditions and often stalls, leading to toxic buildup. For example, Xu et al. reported that 1,1,1-trichloroethane (1,1,1-TCA) and chloroform significantly inhibit reductive dichlorination mediated by *Dehalococcoides* strains carrying the vcrA or tceA genes at concentrations as low as 20 ​μmol/L. This inhibition impedes the critical transformation of vinyl chloride (VC) to ethene, thereby leading to substantial VC accumulation in the environment [5]. Similarly, PCB dechlorination frequently interrupts at mono- and di-chlorinated biphenyls [6]. Anaerobic defluorination of short- to medium-chain PFAS moves slowly and does not completely occur, which leads to persistent fluorinated intermediates [7]. Moreover, reductive defluorination—a process that poses considerable challenges due to the exceptional stability of the C–F bond—has been observed under anaerobic conditions in certain PFAS, including specific C5 unsaturated fluorinated carboxylic acids (C5-PFCA) [8,9].

In contrast, aerobic oxidative dehalogenation targets low-halogenated substrates using oxygenases to initiate ring cleavage. While capable of mineralization, as seen with a resuscitated *Streptococcus* strain SPC0, which facilitates co-metabolic dehalogenation through oxidative mechanisms and achieves complete mineralization of PCBs via the protocatechuate pathway [10], aerobic cometabolism of PCBs can also deliver persistent chlorinated acetophenones [11]. Critically, the removal of halogen atoms makes the water-soluble HOPs degradation products more mobile and enhances their leaching to groundwater and contamination zones. This also improves bioavailability and raises exposure risks for aquatic species and humans. Substantial evidence supports these concerns: in vitro studies indicate aryl hydrocarbon receptor activation and genotoxicity at low concentrations, while microcosm and field studies link intermediates to invertebrate reproductive failure and regulatory exceedances [12].

## 3 Bottlenecks as a design challenge: The case for engineered solutions

The failures described above are not random but arise from identifiable bottlenecks related to microbial community structure, enzyme specificity, and environmental sensitivity. Traditionally, the focus has been on optimizing these factors in situ. However, their inherent complexity and nonlinear interactions make this approach unreliable for preventing the detoxification paradox. We contend that these bottlenecks should be reframed as critical design criteria for the development of next-generation bioremediation technologies.

The effectiveness and safety of microbial dehalogenation rely on the complex interactions. Complete detoxification requires functional microbial consortia with the presence of key organisms, such as certain *Dehalococcoides* strains, to avoid accumulating carcinogenic intermediates like vinyl chloride. Syntrophic interactions and balanced electron donor supply are essential but easily disrupted [13]. In regard to metabolic enzymes, reductive dehalogenases and oxygenases exhibit strict regioselectivity toward limited substrate scopes, leading to persistent or toxic products when certain congeners are preferred. Environmental factors, such as low redox potential, pH neutrality, and adequate availability of toxins and nutrients, are vital for sustaining catalytic activity and microbial fitness [3].

This vulnerability to biotic and abiotic disturbances further elaborates on the limitations of a passive, ecology-centric approach. Rather than attempting to fine-tune inherently unpredictable natural systems, the field should shift toward engineering robust and controllable biological systems. The following section outlines the proposed framework, which directly addresses these bottlenecks through integrated design at the molecular, cellular, and system levels.

## 4 A multi-level engineering framework for genuine detoxification

Addressing the “detoxification paradox” requires a fundamental shift from passive observation to active biological design, with advanced strategies emerging across molecular, cellular, and systems levels to steer degradation pathways toward genuine detoxification.

**1)** **Molecular level: Engineered precision**

Enzyme engineering through directed evolution and rational design offers a means to overcome the inherent limitations of natural enzymes. The objective is to develop biocatalysts with expanded substrate specificity, modified regioselectivity toward recalcitrant congeners, and improved stability under operational conditions. Such engineered enzymes can be deployed in immobilized forms or integrated into artificial genetic circuits within chassis organisms to enable precise and complete biodegradation, ensuring targeted reactions proceed to completion.

**2)** **Cellular level: Consortia by design**

To address the fragility of natural microbial communities, we advocate for the use of Synthetic Microbial Consortia (SMCs). SMCs are rationally designed to incorporate specialized strains that carry out complementary metabolic functions, establishing a synthetic food chain for pollutants that prevents the accumulation of toxic intermediates. Stability is engineered through built-in interdependencies, such as obligate syntrophy and quorum-sensing circuits, rendering SMCs more predictable and effective than natural microbial communities.

**3)** **System level: Hybrid synergy**

Hybrid biotic–abiotic systems integrate the advantages of biological and chemical processes to address persistent bottlenecks in environmental remediation. For example, coupling microbial dechlorination with sulfidized nano-zero-valent iron enables the abiotic reduction of recalcitrant intermediates such as vinyl chloride to ethene [14]. Bioelectrochemical systems facilitate precise electron delivery to dechlorinating microorganisms, while advanced oxidation processes can effectively eliminate residual toxic intermediates without compromising the integrity of the core microbial process [15].

Collectively, this integrated framework advances beyond passive observation toward controllable intervention, effectively mitigating the risks of intermediate accumulation and aligning bioremediation outcomes with genuine detoxification.

## 5 Conclusion and future perspectives

The recognition of the “detoxification paradox” has redefined bioremediation, emphasizing that degradation is not synonymous with detoxification. This shift demands a move from empirical methods to predictive and verifiable strategies. A multidisciplinary integration of ecology, enzymology, and engineering is essential to address metabolic bottlenecks that lead to toxic intermediate accumulation. In-depth diagnostics, including metabolite monitoring and functional gene analysis, are needed to enable targeted interventions. Genome-scale metabolic models will be crucial for simulating outcomes and optimizing strategies like bioaugmentation prior to field applications. Future efforts should focus on developing engineered enzymes and stabilized microbial consortia, incorporating real-time biosensing for toxicity validation, and demonstrating integrated solutions at a pilot scale. Ultimately, the goal is to achieve controlled biological systems that ensure not only pollutant removal but also genuine detoxification with an improvement in environmental safety and sustainable remediation.

## CRediT authorship contribution statement

**Qian Wang:** Writing – review & editing, Writing – original draft, Investigation, Formal analysis, Conceptualization. **Shuai Wang:** Writing – review & editing, Formal analysis. **Yueli Hu:** Writing – review & editing, Formal analysis. **Jiaxin Wu:** Writing – review & editing. **Shanyuan Niu:** Writing – review & editing, Supervision, Funding acquisition, Conceptualization.

## Declaration of competing interests

The authors declare no competing interests.
